# Comparison of Nonclassic and Classic Phenotype of Hypertrophic Cardiomyopathy Focused on Prognostic Cardiac Magnetic Resonance Parameters: A Single-Center Observational Study

**DOI:** 10.3390/diagnostics12051104

**Published:** 2022-04-28

**Authors:** Magdalena Stachera, Paweł Przybyło, Katarzyna Sznajder, Marek Gierlotka

**Affiliations:** 1 Clinical Department of Diagnostic Imaging, Institute of Medical Sciences, University of Opole, 45-052 Opole, Poland; katarzyna.sznajder@uni.opole.pl; 2 Department of Cardiology, University Hospital in Opole, 45-401 Opole, Poland; pawel.przybylo@usk.opole.pl; 3 Department of Cardiology, Institute of Medical Sciences, University of Opole, 45-401 Opole, Poland; marek.gierlotka@uni.opole.pl

**Keywords:** magnetic resonance imaging, late gadolinium enhancement, hypertrophic cardiomyopathy, phenotype, sudden cardiac death, new imaging techniques, prognosis, outcomes, functional imaging, left ventricular obstruction, left atrium, mitral valve apparatus

## Abstract

Patients with nonclassic phenotypes (NCP)—more advanced stages of hypertrophic cardiomyopathy (HCM)—constitute an intriguing and heterogeneous group that is difficult to diagnose, risk-stratify, and treat, and often neglected in research projects. We aimed to compare cardiac magnetic resonance (CMR) parameters in NCP versus classic phenotypes (CP) of HCM with special emphasis given to the parameters of established and potential prognostic importance, including numerous variables not used in everyday clinical practice. The CMR studies of 88 patients performed from 2011 to 2019 were postprocessed according to the study protocol to obtain standard and non-standard parameters. In NCP, the late gadolinium enhancement extent expressed as percent of left ventricular mass (%LGE) and left ventricular mass index (LVMI) were higher, left atrium emptying fraction (LAEF) was lower, minimal left atrial volume (LAV min) was greater, and myocardial contraction fraction (MCF) and left ventricular global function index (LVGFI) were lower than in CP (*p* < 0.001 for all). In contrast, HCM risk score and left ventricular maximal thickness (LVMT) were similar in NCP and CP patients. No left ventricular outflow tract obstruction (LVOTO) was observed in the NCP group. Left ventricular outflow tract diameter (LVOT), aortic valve diameter (Ao), and LVOT/Ao ratio were significantly higher and anterior mitral leaflet (AML)/LVOT ratio was lower in the NCP compared to the CP group. In conclusion, significant differences in nonstandard CMR parameters were noted between the nonclassic and classic HCM phenotypes that may contribute to future studies on disease stages and risk stratification in HCM.

## 1. Introduction

Hypertrophic cardiomyopathy (HCM) is defined in the European guidelines by the presence of increased left ventricular (LV) wall thickness, which is not solely explained by abnormal loading conditions [1], with no assumptions about etiology or myocardial pathology. This definition aligns with everyday clinical practice and was accepted for this paper, though it differs substantially from the American guidelines [2]. The common perception and natural history of HCM have significantly changed recently. In the past, HCM was widely considered to be a disease with an unfavorable prognosis (an annual mortality between 4% and 6%) and most of the information used to come from only two specialized centers [3]. Now, due to the introduction of new diagnostic and therapeutic methods, particularly cardiac magnetic resonance (CMR), as well as publications from non-selected cohorts from all over the world, the understanding of HCM has improved [3,4]. The treatment methods have become more effective, and in highly specialized centers, the disease-related mortality is currently reported to be as low as 0.5% per year [5].

The most important achievements are more effective risk stratification and the use of the implantable cardioverter defibrillator (ICD) for primary prevention of sudden cardiac death (SCD). It was estimated that only about 25% of patients with HCM ultimately died of their disease, predominantly those who were <30 years of age at presentation [5]. Furthermore, it was noted that the process of life-long left ventricle (LV) remodeling and progressive dysfunction may result in a considerable number of HCM patients as a relatively rare clinical condition described previously as the end-stage or burned-out phase. The phenotypes and stages of the disease were presented in 2012, and the so-called classic phenotype was defined as ejection fraction (EF) > 65%, according to Olivotto et al. [6]. The other phenotypes or stages of the disease included stage III—adverse remodeling (AR) and stage IV—overt dysfunction. In this paper, we grouped stage III and IV together as the nonclassic phenotype (NCP), defined as EF ≤ 65% [6].

It is indicated that certain subgroups of the HCM patients face a worse prognosis: the patients with a high risk of SCD with no symptoms of heart failure, those with progressive symptoms of congestive heart failure (CHF) with or without left ventricle outflow tract obstruction (LVOTO) in the absence of systolic dysfunction, and the so-called end-stage disease characterized by LV systolic dysfunction or apical aneurysms. The development of atrial fibrillation (AF) has an unfavorable hemodynamic impact on the clinical course of HCM and is associated with a significant risk of thromboembolism and stroke [3,4,7]. CHF has become an important cause of morbidity and mortality in patients with HCM, being responsible for as many as 60% of disease-related deaths [3,4,5,8]. Implantable cardioverter defibrillator (ICD) implantation is unequivocally indicated for secondary prevention of SCD [1,2,9], while indications for primary prevention are much more ambiguous. CMR gives new perspectives in HCM patient prognostication. Therefore, numerous parameters were proposed.

The assessment of an individual patient’s risk for SCD is nuanced and actively being refined; it continues to evolve as new imaging prognostic markers emerge (e.g., apical aneurysm, decreased left ventricular systolic function, and extensive late gadolinium enhancement (LGE)), with some variability in recommendations from different society guidelines [1,2,10]. The need for a new modified HCM risk score seems to emerge.

The aim of our study was the comparison of CMR parameters of nonclassic versus classic phenotypes of HCM with special emphasis given to the markers of established and potential prognostic importance, based on the CMR studies of the patients referred to our center, mostly outpatients. It was a single-center observational study in the “real world” setting. To the best of our knowledge, this was the first comparison of prognostic CMR parameters in the population of HCM patients grouped according to the nonclassic versus classic phenotype.

## 2. Materials and Methods

### 2.1. Patients

The investigated group consisted of 88 patients referred for CMR in our hospital from 2011 to 2019. The inclusion criteria were as follows: LV wall thickness ≥15 mm or ≥13 mm with family history of HCM, according to the 2014 European Society of Cardiology (ESC) Guidelines [1]. The exclusion criteria were as follows: amyloidosis apparent during CMR study and severe aortic stenosis. Images from representative CMR studies are available in [11].

According to Olivotto et al., the definitions of the HCM disease stages are as follows: stage I: nonhypertrophic HCM, the absence of LV hypertrophy in individuals with known HCM-causing mutations; stage II: the “classic” HCM phenotype when the LV is hyperdynamic (as defined by an ejection fraction [EF] > 65%), stage III: adverse remodeling, the presence of unfavorable structural modifications, superimposed to the “classic” HCM phenotype with LVEF in the low to normal range of 50% to 65%, and stage IV: overt dysfunction defined by an LVEF < 50% [6].

For the purpose of our study, the nonclassic phenotype (NCP) was defined as ejection fraction (EF) ≤ 65%, and classic phenotype (CP) as EF > 65% [6].

### 2.2. Image Acquisition and Analysis

All the CMR studies were performed on a 1.5 T scanner (Avanto, Siemens, Erlangen, Germany) using the standard protocol. Long- and short-axis stack cine images were acquired for assessment of myocardial function. The routine LGE imaging included a breath-hold segmented inversion recovery sequence covering the entire myocardium obtained 10 min after administration of a commercially available gadolinium-based contrast agent at a standard dosage (0.1 mmol/kg), with the inversion time adjusted to null the normal myocardium.

All the CMR studies were postprocessed according to the study protocol with a dedicated software, i.e., *syngo.via* MR Cardiac Analysis (Siemens Healthineers). The left ventricular semi-automated segmentation algorithm included the papillary muscles and trabeculae in the blood pool, and excluded them from the left ventricular mass. The LV contours were corrected by the investigator (M.S.) when appropriate. Apart from routine LV functional parameters and mean segmental thickness, volume–time curves were derived automatically by the software for the estimation of the following: PER (peak ejection rate), PET (peak ejection time), PFR (peak filling rate) and PFT (peak filling time). The maximal wall thickness of the left and right ventricles (LVMT and RVMT) were determined in each patient in short-axis slices manually.

Myocardial contraction fraction (MCF) and left ventricular global function index (LVGFI) were calculated manually according to the formulas given in [12], with parameters obtained from routine non-contrast CMR sequences. MCF is defined as the ratio of stoke volume divided by myocardial volume [13] and LVGFI as the ratio of stroke volume divided by LV total volume (the sum of mean LV cavity and myocardial volumes) [14].

LGE areas were quantitated in all of our patients as LGE volume and as a percentage of the total LV mass with the manual visual method included in the analysis package. Segmental distribution of LGE was also noted.

Left atrium (LA) endocardium was traced manually and LA long axis was measured in standard 4-chamber and 2-chamber cine long-axis acquisitions, excluding the left atrial appendage and pulmonary veins. Parameters were taken at the end of ventricular systole (maximal) and when the size of the chamber was minimal by visual assessment. Additionally, LA volumes were calculated manually by the biplane area method as in [15,16]. LA parameters were expressed as LAVmax (left atrial maximal volume), LAVmin (left atrial minimal volume), LAEF (left atrial emptying fraction), and LAVI (left atrial maximal volume index). LAEF was calculated as (LAVmax − LAV min)/LAVmax [15].

Left ventricle outflow tract (LVOT), aortic valve diameter (Ao), anterior mitral leaflet (AML), posterior mitral leaflet (PML), and LA 3-chamber (LA 3Ch) dimensions were measured manually from routine cine three-chamber images, as in [17,18,19]. LA 3Ch dimension was taken analogously as in echocardiographic examination. LVOT velocity was recorded in patients with resting LVOTO using phase-contrast through-plane and in-plane sequences and quantitated by flow velocity calculation software when applicable.

### 2.3. Statistical Analysis

Quantitative variables are expressed as median (interquartile range) and the differences between the groups were tested by the Mann–Whitney U tests. For correlation calculations, Spearman’s rank correlation coefficient was used. Chi-square Pearson tests (with Yates correction where appropriate) were used for categorical variables. The *p* value < 0.05 was considered as statistically significant. Statistical analysis was performed using Statsoft Statistica 13 software (TIBCO Software, Palo Alto, CA, USA) and R statistical software (R Foundation for Statistical Computing, Vienna, Austria).

## 3. Results

### 3.1. The Study Population

**Figure 1:**
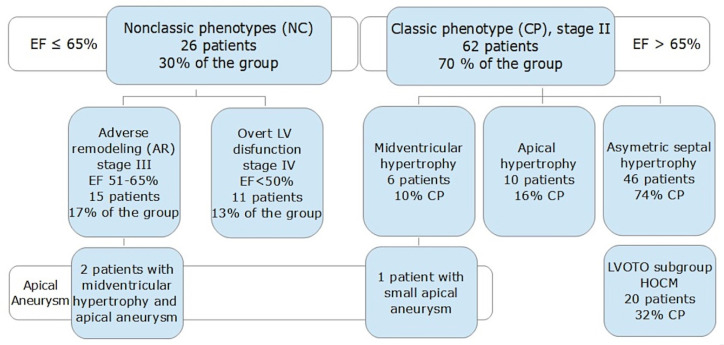
HCM phenotypes and stages in the study group according to Olivotto et al., 2012 [6].

**Figure 2:**
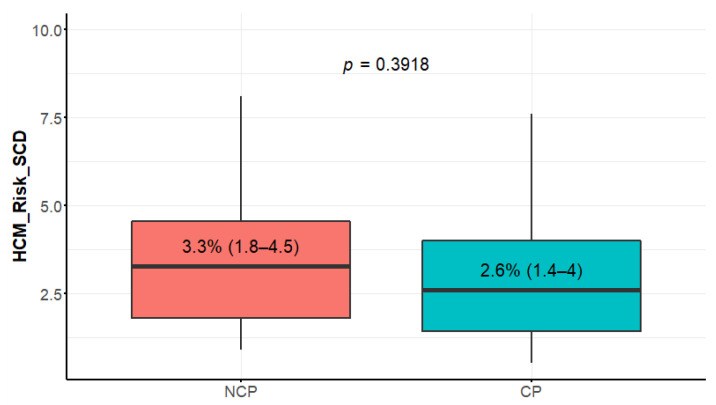
HCM risk score (hypertrophic cardiomyopathy European Society of Cardiology risk score) in NCP (nonclassic phenotype) and CP (classic phenotype) patients. Data presented in boxplot as median values, interquartile range, and minimal and maximal values.

The study group consisted of 88 patients (median age 60, IQR 56–68, range 29–80 y/o), and 61% of them were men. A total of 62 (70%) of the patients had EF > 65% and were classified as CP, while NCP presented in 26 of patients with EF ≤ 65% (Figure 1). The results of the comparison of NCP and CP are presented in Table 1. No significant differences in the HCM risk score [1] were observed between the groups (Figure 2). Both LVMT and indexed maximal wall thickness (IMWT) were similar in the NCP patients and the CP patients.

### 3.2. Left Ventricular Aneurysm

There were two patients with ventricular aneurysms and midventricular hypertrophy in the NCP group and only one patient with a notably small aneurysm in CP (Figure 1). No apical aneurysm was detected by echocardiogram. Representative images of these cases are available in [11].

Significant differences were noted regarding the following CMR parameters.

### 3.3. Late Gadolinium Enhancement Extent

**Figure 3:**
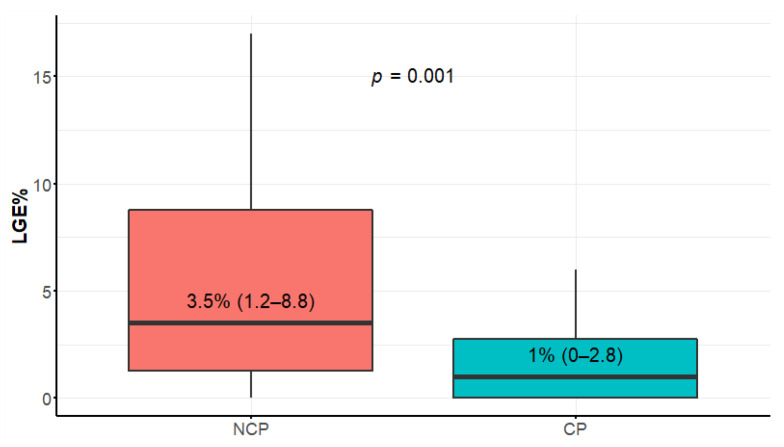
LGE (late gadolinium enhancement) extent comparison between nonclassic and classic phenotypes. Data presented in boxplot as median values, interquartile range, and minimal and maximal values.

**Figure 4:**
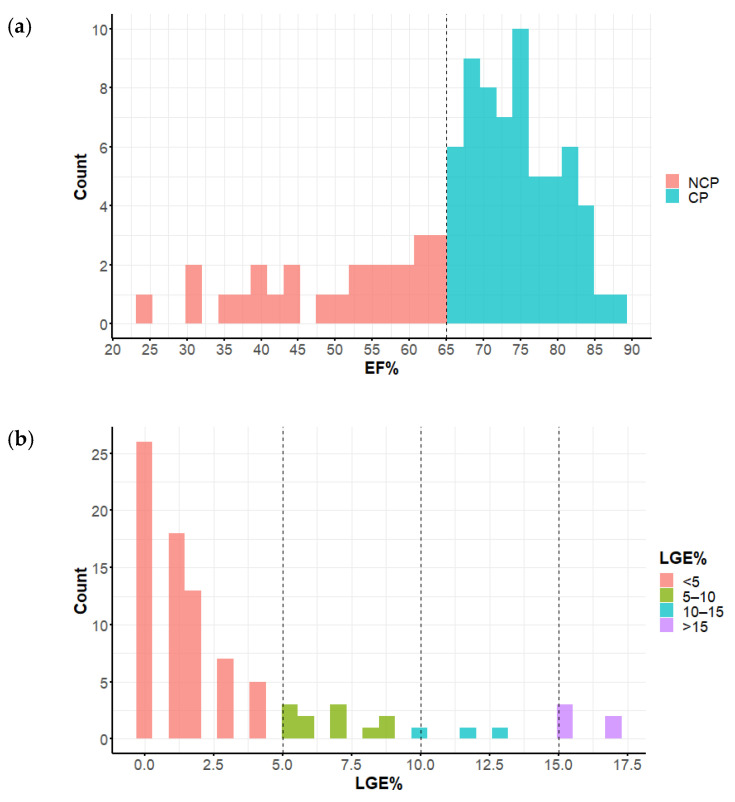

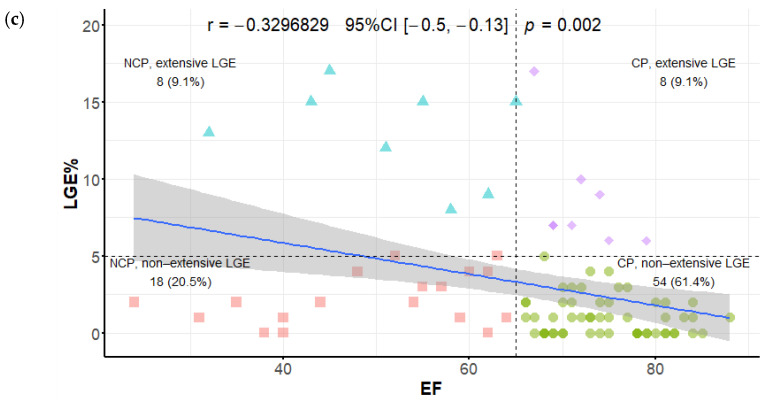
(**a**) EF (ejection fraction) distribution in the study group, EF = 65% was the cutpoint splitting the study subgroups into NCP (nonclassic phenotype) and CP (classic phenotype); (**b**) quantitative LGE (late gadolinium enhancement) distribution among the study groups. (**c**) Correlation of EF and LGE% (late gadolinium enhancement expressed as the percent of myocardial mass); LGE% = 5 and EF = 65% split the patient group into four quarters.

**Figure 5:**
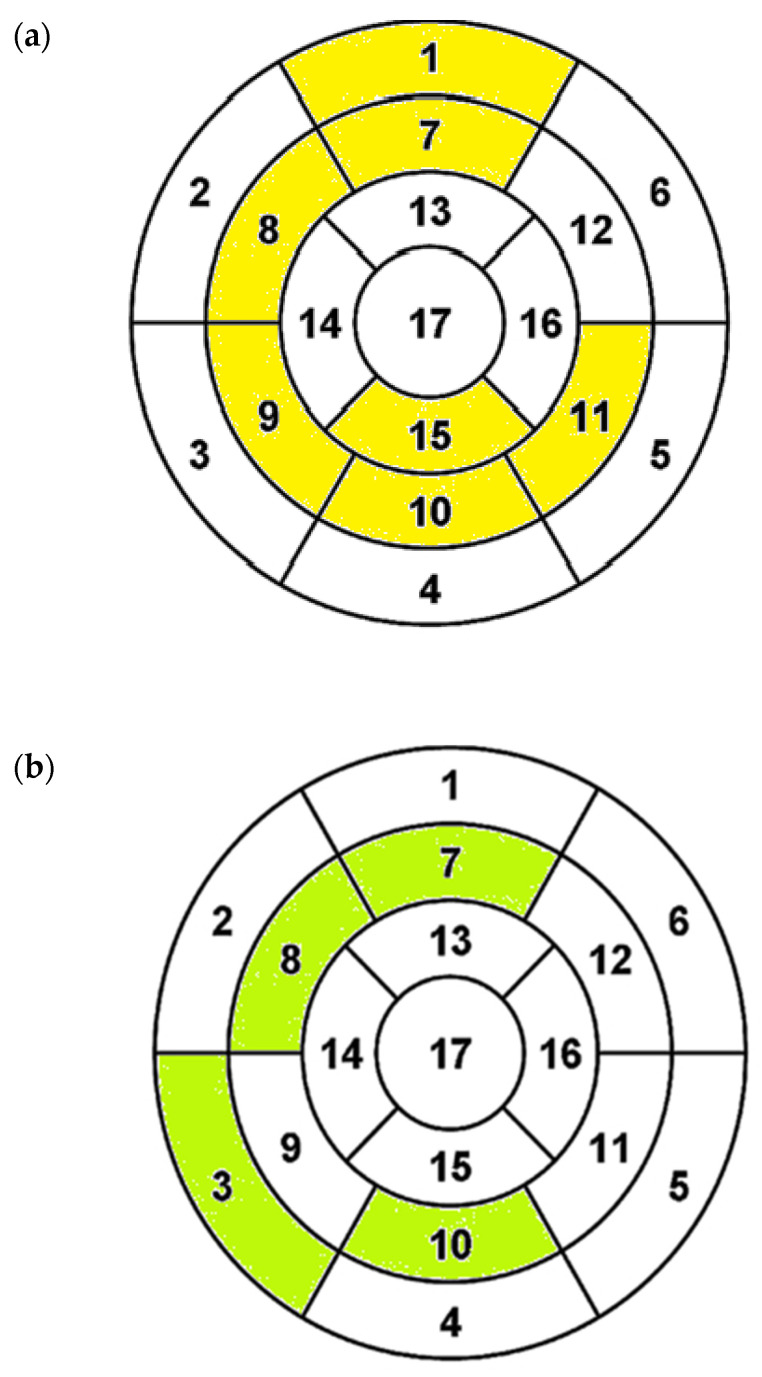
(**a**) Segmental myocardial thickness—in NCP (nonclassic phenotype), greater mostly in midsegments (marked as yellow); (**b**) segmental LGE (late gadolinium enhancement) distribution. The usual locations of LGE areas in HCM (hypertrophic cardiomyopathy) were insertion points of the right ventricle and focal areas in hypertrophied regions. In the NCP group, the enhancement was more frequent beyond the insertion points, indicating more extensive distribution (marked as green).

Compared to CP, the NCP patients had significantly more LGE% (Figure 3), and the difference was even more striking in LGE volume (Table 1). The distributions of EF and LGE% as well as the correlations of these parameters are given in Figure 4a–c. Most of the patients belonged to the lowest range of LGE (0–5%), and only few belonged to the highest range > 15% (“extensive LGE” by Chan et al. [20]). EF and LGE% were only modestly negatively correlated (r = −0.33). LGE% = 5 and EF = 65% split the patient group into four quarters with equal numbers of patients with LGE > 5% in NCP and CP groups (“extensive LGE” by Greulich et al. [9]). LGE segmental distribution was different in the study groups—in NCP patients, the areas of late enhancement were more frequent in segments 3, 7, 8, and 10 (beyond insertion points)—see Table 2 and Figure 5.

### 3.4. LV Mass Index

The median left ventricular mass index (LVMI) in the NCP group was 111 g/m^2^ (IQR 101–150) compared to 93 g/m^2^ (IQR 80–113) in the CP group, *p* < 0.0001. The LV segmental thickness was greater in segments 1, 7–11, and 15 (mostly midsegments)—see Table 2 and Figure 5a.

### 3.5. Myocardial Contraction Fraction (MCF) and Left Ventricular Global Function Index (LVGFI) 

**Figure 6:**
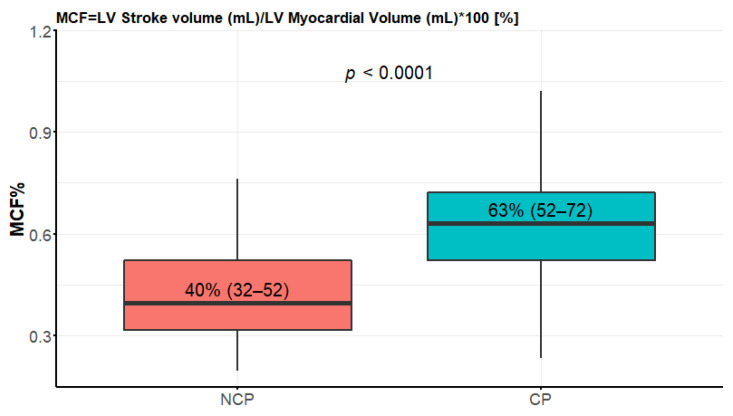
Myocardial contraction fraction comparison between nonclassic and classic phenotypes. Data presented in boxplot as median values, interquartile range, and minimal and maximal values.

The NCP patients had significantly lower values of MCF (Figure 6). Both groups had values much lower than the reference given in [21], that is, 136.3 ± 24.4%. The median LVGFI in the NCP group was 26 (IQR 19–31) compared to 41 (IQR 36–45) in the CP group, *p* < 0.0001.

### 3.6. Left Atrial Parameters

**Figure 7:**
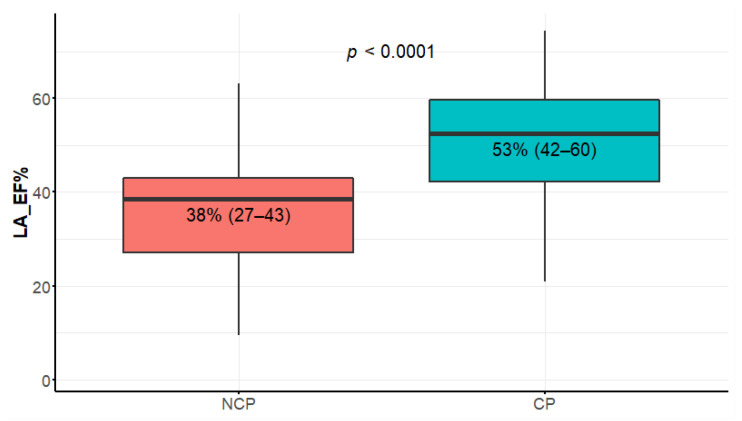
LA EF% (left atrial emptying fraction) comparison between nonclassic and classic phenotypes. Data presented in boxplot as median values, interquartile range, and minimal and maximal values.

The differences in LAVmax, LAVI, and LAA between the NCP and CP patients were not statistically significant. In contrast, the LAEF difference was strikingly significant (Figure 7). The minimal volume (LAV min), area (LAA), and length (LAL) atrial parameters were also significantly different (Table 1).

### 3.7. Volume–Time Curve Parameters

In the NCP patients, a longer peak ejection time (PET) was observed: median 128 ms (IQR 114–149) versus 110 ms (IQR 91–125) in CP, *p* = 0.0012, probably due to impaired systole in nonclassic phenotypes. The peak ejection rate (PER), peak filling rate (PFR), and peak filling time (PFT) differences did not reach statistical significance (Table 1). On the other hand, normalized peak filling rate in NCP differed significantly: PFR/LVEDV was shorter in NCP patients, and PFR/SV was longer, likely reflecting diastolic dysfunction (Table 1).

### 3.8. Right Ventricular Involvement

The right ventricular maximal thickness (RVMT) appeared greater in NCP—median value of 4.5 mm (IQR 3–6) versus 3 mm (IQR 2–4), *p* = 0.0013—probably due to more advanced disease.

### 3.9. Left Ventricular Outflow Tract and Mitral Apparatus

**Figure 8:**
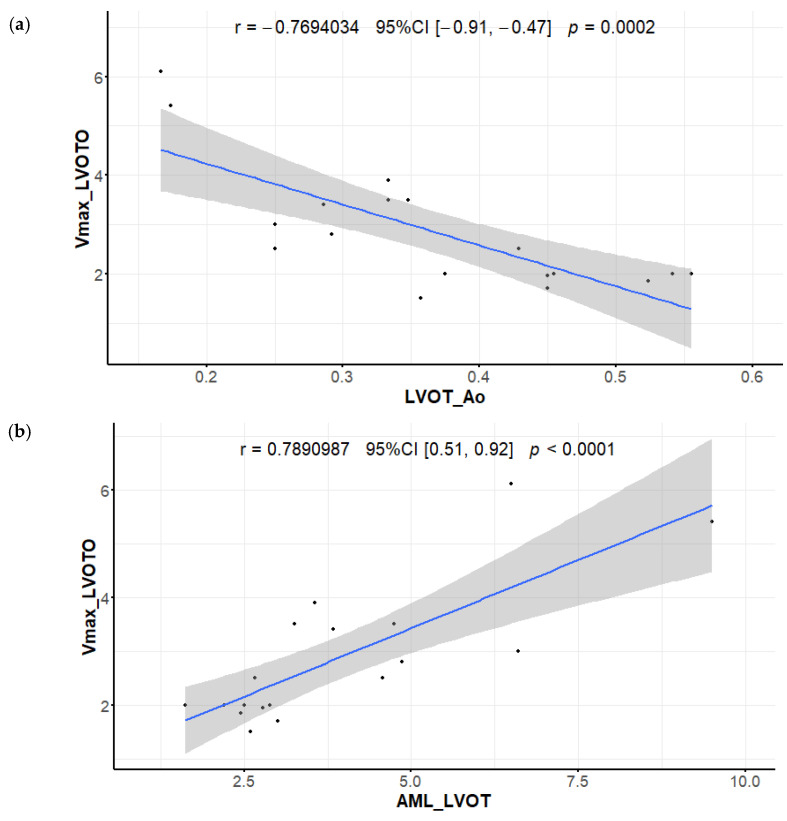
Correlation between maximal velocities measured in left ventricular outflow tract (m/s) and (**a**) the LVOT/Ao (left ventricular outflow tract diameter/aortic valve diameter) ratio; (**b**) the AML/LVOT (anterior mitral leaflet length/left ventricular outflow tract diameter) ratio.

No LVOTO was observed in our NCP group, as a result of progressive LV remodeling. Consequently, LVOT, Ao, and LVOT/Ao were significantly higher, and AML/LVOT was lower in NCP (Table 1). Good correlations between LVOT velocity and LVOT/Ao as well as between LVOT velocity and AML/LVOT were found (Figure 8).

## 4. Discussion

According to the available data, this was the first report comparing advanced CMR parameters, not routinely used, in nonclassic phenotype (NCP) and classic phenotype (CP) groups of HCM patients. A comprehensive profile of the disease stages may facilitate optimal management and better prognostication [3,6,22]. On the one hand, primary prevention ICD placement indications are evolving, including HCM patients with EF < 50%—stage IV, overt dysfunction [2,23]—as a high rate of appropriate ICD was noted in this group. On the other hand, a rapid CHF progression is not inevitable in nonclassic phenotypes, and some patients benefit from long periods of clinical stability [24,25]. An increased risk of progressive decline was observed recently not only in stage IV (overt LV dysfunction) but also in the HCM patients with LVEF below 60–65% in stage III (adverse remodeling) [4,24,25,26], and because of this and the relatively lower numbers of patients, we decided to group nonclassic phenotypes together.

Our results indicated the significant differences in quantitative LGE, LVMI, and LA parameters, MCF and LVGFI, between the nonclassic and classic HCM phenotypes. We believe this may be useful in clinical practice. We assumed that if numerous parameters are more frequent in advanced stages of the disease, it is likely that they reflect progression of heart dysfunction and may help in clinically relevant staging of the disease. Due to the complex pathological mechanisms involved, new prognostic parameters, beyond EF, seem to be promising in risk stratification and deserve further prospective evaluation. Of note is that a significant proportion of research on HCM risk stratification concentrates on CP patients only.

### 4.1. HCM Risk Score, LVMT, and Indexed Maximal Wall Thickness (IMWT)

HCM risk score was retrospectively developed and first published in 2014 [27]; it is incorporated into ESC guidelines [1]. Many studies validated this model. In 2019, a meta-analysis was published [28], concluding excellent specificity, poor sensitivity, and moderate discrimination performance. The data on the prevalence of NCP in study populations are difficult to elucidate. It was surprising that no significant differences in risk score occurred between the classic phenotype and the more advanced stages in our study. Since the risk of cardiac events in stage four patients is estimated to be around 8% [29], it seems that the HCM risk score in NCP patients is inadequate. The results of our study indicated that the new 2020 AHA/ACC guidelines [2], advising ICD implantation in end-stage disease, might be more adequate for these patients than the 2014 ESC risk score methodology.

LVMT is an important classic prognostic parameter analyzed in [30] and incorporated into the ESC HCM risk score. In our study, there was absolutely no difference in median LWMT between the study groups. We believe it might reflect LV remodeling in stages III and IV with progressive thinning of the myocardium as the chamber dilates despite the more advanced disease. CMR is important for adequate maximal wall thickness LVMT measurement, which is clinically relevant especially at diagnostic (15 mm) or prognostic (30 mm) cutoffs [10,31].

### 4.2. Left Ventricular Apical Aneurysm

There was only one notably small aneurysm in our CP group. However, two aneurysms were detected in stage III—adverse remodeling, and as usual, they were a consequence of LV adaptation to progressive midventricular obstruction. It was shown retrospectively that the HCM patients with LV apical aneurysms, developed in the evolution of midventricular obstruction phenotype, are at high risk for arrhythmic sudden death and thromboembolic events [2,10,32]. Fortunately, none of our patients developed a thrombus in the aneurysm.

### 4.3. LGE Extent

LGE reflects the focal distribution of fibrosis in HCM [22]. There is accumulating evidence based on numerous well-documented retrospective and prospective studies as well as the meta-analysis including thousands of patients that the extent of LGE may identify individuals at higher risk of SCD and predict the development of end-stage HCM with systolic dysfunction. Conversely, the absence of LGE was associated with lower risk for SCD events [9,20,33,34]. The extent of LGE has been recognized as a prognostic parameter since 2014 and was included in the new 2020 AHA/ACC guidelines. On the contrary, the ESC risk score had been developed before the results of studies with sufficient statistical power emerged.

Unfortunately, a number of practical challenges prevent the routine use of LGE quantification in everyday clinical practice. The differences in CMR scanners, LGE sequences, contrast doses and types, inversion times, and software preclude universal agreement [10,35]. Chan et al. [20] pointed to the value of LGE ≥15% of LV mass as extensive. In a recent study, Greulich et al. [9], after a 10 year follow-up, concluded that HCM patients with >5% LGE might be candidates for primary SCD prevention and should be carefully monitored for progression. Different methods of LGE quantification were used in the above mentioned studies (≥6 SDs in [20] and ≥2 SDs in [9]).

Our study showed that 68% of our patients belonged to the lowest range 0–5%LGE (cf. Figure 1) and only 3% to the range ≥ 15% LGE. In such a situation, the 5% cutpoint seems more practical. Since we used a different method of LGE quantitation, it is not clear to what extent the results of the above-mentioned studies are applicable to our population. This is an extremely important issue for everyday practice.

### 4.4. LV Mass Index

Left ventricular mass index is a simple, routine, and well-known CMR parameter. Correlation with HCM-related mortality was demonstrated in the 2008 retrospective study by Olivotto et al. [30], and the cutpoints of 91 g/m^2^ for men and 69 g/m^2^ for women were suggested. In a recent retrospective study from Hungary, LVMI was confirmed as an independent predictor of major events with slightly different cutoffs (108 g/m^2^ for men and 86 g/m^2^ for women) [36]. In our study, the difference between CP and more advanced stages was strikingly significant (*p* < 0.001) and more myocardial segments were hypertrophied, mostly midsegments. In our opinion, it reflected the pattern of disease progression.

### 4.5. Myocardial Contraction Fraction (MCF) and Left Ventricular Global Function Index (LVGFI) 

A promising novel prognostic CMR parameter is myocardial contraction fraction (MCF). MCF was first described by King et al. [13] as the volumetric measure of myocardial shortening in left ventricular hypertrophy [21], defined as the ratio of stroke volume (SV) to left ventricular myocardial volume (LVMV), measured from freehand three-dimensional echocardiography. MCF has been suggested as a useful biomarker in the diagnostic workup of the patients with left ventricular hypertrophy [13,21]. The MCF demonstrates that myocardial performance is decreased in hypertensive hypertrophy and increased in physiologic hypertrophy. It may be useful in assessing patients with similar degrees of myocardial thickening [13]. MCF has been shown to be a valuable prognostic factor in healthy people and multiethnic populations [37,38], patients with aortic stenosis (AS) undergoing transcatheter aortic valve replacement (TAVR) [39], patients with cardiac amyloidosis [40,41,42], and patients with non-ischemic dilated cardiomyopathy [43].

The values of MCF for healthy people are given in many population-based and clinical studies [12,13,21,37,38,43,44], and the differences, sometimes striking, probably stem from various modalities (echo vs. CMR), methodology, and selection bias.

Lower MCF can indicate worsening of LV function with preserved ejection fraction because of a reduction in ventricular capacitance, especially in hypertrophied ventricles [44].

The researchers from Switzerland [21] suggested the cutoff of MCF < 50% and EF < 60% as identifying patients with high probability of cardiac amyloidosis in the study of patients with different etiologies of hypertrophy. It is important to note that in [21], only the patients with CP of HCM were evaluated. In contrast, our results indicate that MCF < 50% was a usual finding in the NCP HCM phenotype in our population.

In echocardiography-based single-center studies of MCF in HCM patients published in 2019 and 2020 [45,46], MCF was significantly associated with functional capacity, adverse cardiovascular outcomes, and mortality. Only the patients with EF > 60% and EF ≥ 55% were evaluated in [45,46], respectively, so the NCP population was not adequately represented. This and the relatively small number of patients preclude the routine application of the results. The CMR method of MCF estimation seems superior to echo because it is more accurate and reproducible [21], as echocardiography introduces error due to the asymmetric nature of LV hypertrophy in HCM [44,45].

The median MCP in our study was significantly below normal CMR values in both CP and NCP groups and it seemed to be a sensitive marker of pathological hypertrophy. The difference between CP and more advanced stages was also distinct; therefore, we believe that the MCP may prove to be particularly useful for HCM progression monitoring, reflecting the dysfunction in NCP patients, since it is considered to be a measure of myocardial shortening [38].

Left ventricular global function index (LVGFI) is another novel prognostic parameter introduced in [14], derived from the standard cine CMR sequence, as a marker for assessment of cardiac performance. It reflects the alterations in LV mass and cavity size in the process of LV remodeling [12], involving both SV and the total heart size [14]. To the best of our knowledge, it was never evaluated in the prognostic context in HCM patients.

In our study, there was a clear and significant difference between the groups, and we believe LVGFI might be well-suited to reflect the specific nature of LV remodeling in HCM. It would be interesting to evaluate LGFI in a properly designed and powered prognostic study as well as apply it retrospectively to patients from important registries, e.g., the Sarcomeric Human Cardiomyopathy Registry (SHaRe) or an ongoing HCM registry.

In a recent paper by Huang S et al. [12], LVGFI was suggested for differentiating cardiac amyloidosis from hypertrophic cardiomyopathy; it included only CP patients in the HCM group. It suggested cutoffs of 39.1 and 37.4 as specific for amyloidosis. In our study, however, the NCP group’s median value of LVGF was 26 (19–31); therefore, all of our NCP patients would be classified as amyloid according to criteria given in [12].

CMR-derived MCF and LGFI are promising parameters; however, further studies are needed to verify the clinical utility in HCM, with emphasis on including the NCP HCM population.

### 4.6. Left Atrial CMR Parameters

Left atrial CMR parameters in the context of HCM have been frequently studied in recent years [15,16,47,48]. Left atrium (LA) enlargement is well-known prognostic factor, included in the ESC HCM risk score [1]. Importantly, it is a three-dimensional structure, and can be inconsistently measured on two-dimensional echocardiography [10]. In the asymptomatic population of the Multi-Ethnic Study of Atherosclerosis (MESA study), the CMR-assessed elevated LA volumes and decreased LA emptying fractions were independently associated with incident atrial fibrillation (AF) [49]. AF is the most frequent arrhythmia in patients with HCM, and its prevalence ranges between 20% and 30% [50]. It is an important determinant of clinical deterioration due to heart failure or embolic stroke. 

The CMR measures of LA remodeling and dysfunction reliably identified the patients with HCM at risk for future development of AF; in [15], multivariate analysis identified LA emptying fraction (LAEF < 38%), LA end diastolic volume (LAEDV ≥ 118 mL), and age (≥40 years) as independently associated with AF occurrence. LAEF constitutes a strong novel CMR marker in this disease [15]. LA dysfunction is also detectable by CMR in preclinical HCM mutation carriers (stage I of the disease), though LA function appears most impaired in subjects with overt HCM and a greater extent of LV fibrosis [16]. It was suggested that the CMR-derived LA indices may improve the contemporary risk stratification algorithms in children with HCM [51]. Since LA dysfunction is prognostically important for AF development, we suppose it may also trigger CHF progression. Artificial intelligence (AI)-enhanced software allows much faster and easier assessment of atrial volumes, hopefully making it practical in everyday clinical routine in the near future [47,51,52]. Novel CMR markers of LA performance probably reflect mostly diastolic dysfunction and may potentially prove useful for disease staging [35,47]. Our study underlined the value of LAEF and minimal LA volume and area as more sensitive than the maximal parameters. The echo-derived minimum left atrial volume was previously found to be more sensitive than the maximum left atrial volume also in [53]. The question arises if reporting of minimal LA parameters, though time-consuming, should become standard.

It would be interesting to analyze the prognostic potential of LAEF and LA not only in relation to AF but also to CHF progression, stroke, and combined cardiovascular endpoints.

### 4.7. Volume–Time Curve Parameters

The practical application of these parameters is difficult [11,54], partly because of inconsistent values of normal ranges provided by different authors.

In our study, a longer peak ejection time (PET) was observed in the NCP patients, probably due to impaired systole in nonclassic phenotypes. The normalized peak filling rate in NCP differed significantly, most likely reflecting diastolic dysfunction.

### 4.8. Right Ventricular Involvement

Right ventricular involvement in our NCP patients was greater than in CP, probably because of more advanced disease. A weak positive association between RV hypertrophy and the estimated probability of SCD at 5 years (rho = 0.16, *p* = 0.01) was observed in [55].

### 4.9. Left Ventricular Outflow Tract and Mitral Apparatus

Abnormalities of the mitral apparatus, especially redundant mitral valve leaflets, have been described in HCM, and their importance in the pathogenesis of left ventricular outflow tract obstruction (LVOTO) is appreciated [17,18,56]. The ratio of anterior mitral leaflet (AML) length to LV outflow tract diameter of >2.0 (AML/LVOT) was associated with subaortic obstruction [18]. Another CMR parameter proposed as an accurate marker for LVOT obstruction is the left ventricular outflow tract/aortic valve diameter (LVOT/Ao) ratio [19].

Our study showed that simple CMR parameters derived from routine three-chamber cine images (LVOT/Ao and AML/LVOT) are valuable for resting LVOTO assessment, even without dedicated flow sequences that prolong imaging duration.

LVOTO disappearance is an important part of the disease progression in HCM; as LV remodeling develops, LVOT increases. In our NCP patients, no LVOTO was detectable.

Recently in [57], it was shown that the spectrum of mitral leaflet length and mobility that affects subaortic obstruction also influences mitral regurgitation.

To conclude, we believe that the various parameters investigated in our study may contribute to redefining the classification of HCM stages so that it is better suited for clinical practice and risk stratification purposes. If numerous parameters are more frequent in advanced stages of the disease, it is likely that they reflect pathophysiology of heart chamber remodeling in HCM leading to increasing dysfunction, and may serve as an adjunct to clinical staging and decision making.

Our study also illustrated the notion that the frequent exclusions of the NCP patients from the HCM population in numerous research studies may lead to incorrect conclusions. We believe this population is a challenge in clinical practice.

### 4.10. Limitations

This study was a single-center observation study dealing with a relatively small number of patients. The small numbers of deaths and clinical adverse events (mentioned in our review paper [11]) precluded their statistical analysis. No genetic testing was available. Since the clinical information was missing for a substantial proportion of the outpatient group, it was not considered in this paper. The clinical characteristics of the patients (available data) is presented in Supplementary Material in Table S1, with the partial follow-up results in Table S2.

## 5. Conclusions

Our results showed significant differences in validated and novel prognostically important CMR parameters between the nonclassic and classic HCM phenotypes that may contribute to future studies on risk stratification in HCM. CMR-derived MCF and LGFI were promising parameters; however, further studies are still needed to verify their clinical utility in HCM, with the emphasis on including the NCP HCM population.

## Figures and Tables

**Table 1:** Characteristics of the study groups.

Parameter	NCP	CP	All Patients	*p* Value
Number of patients, *n*	26	62	88	
age (years)	60 (56–68)	61 (46–70)	60 (52–70)	0.9054
BSA (m^2^)	2.0 (1.9–2.2)	1.9 (1.8–2.1)	1.9 (1.8–2.1)	0.0350
BMI (kg/m^2^)	29 (25–33)	29 (26–31)	29 (26–31)	0.8370
HCM risk score (%)	3.3 (1.8–4.6)	2.6 (1.4–4.0)	2.9 (1.5–4.2)	0.3918
Male sex, *n* (%)	21 (80%)	33 (53%)	54 (61%)	0.02912
**LV CMR parameters**				
LVEDV (mL)	172 (153–257)	140 (126–166)	150 (128–179)	0.0005
LVESV (mL)	86 (61–144)	38 (27–47)	44 (30–64)	<0.0001
SV (mL)	93 (72–104)	105 (92–121)	102 (88–116)	0.0042
CO (L/min)	7 (5–7)	7 (5–7)	7 (5–7)	0.7316
LV mass ((g)	241 (197–309)	174 (149–210)	194 (157–243)	0.0009
LVMT (mm)	20 (17–23)	20 (17–22)	20 (17–22)	0.54
**Right Ventricle**				
RVMT (mm)	4.5 (3–6)	3 (2–4)	3 (2–5)	0.0013
**Volume–time curve**				
PER (mL/s)	500 (370–563)	479 (428–605)	491 (428–586)	0.3800
PET (ms)	128 (114–149)	110 (91–125)	115 (100–131)	0.0012
PFR (mL/s)	360 (290–438)	362 (305–437)	362 (299–438)	0.6706
PFT (s)	521 (475–690)	565 (490–875)	559 477–801)	0.0924
**Late Gadolinium Enhancement**				
LGE% (%)	3.5 (1.2–8.8)	1 (0–2.8)	2 (0–4)	0.0010
LGE volume (mL)	9 (3–20)	2 (0–5)	3 (1–9)	0.0004
**Indexed (to BMI) and complex parameters**				
LVEDVI	87 (75–120)	75 (65–83)	79 (68–90)	0.0041
LVESVI	39 (29–67)	18 (14–25)	24 (17–31)	<0.0001
LVMI	111 (101–150)	93 (80–113)	98 (83–120)	<0.0001
PFR/LVEDV	2 (1.5–2.5)	2.5 (2,2–3)	2.4 (2–2,9)	0.0004
PFR/SV	3.9 (3.4–4.7)	3.3 (3–3.9)	3.6 (3–4.2)	0.0024
Wall to volume ratio	0.23 (0.17–0.28)	0.27 (0.21–0.31)	0.26 (0.2–0.31)	0.0401
LVEDV/EDM	0.75 (0.65–0.89)	0.79 (0.65–0.93)	0.78 (0.65–0.91)	0.7454
IMWT	10 (9–12)	10 (9–11)	10 (9–11)	0.8227
MCF	40% (32–53)	63% (52–72)	56% (42–68)	<0.0001
LVGFI	26 (19–31)	41 (36–45)	38 (30–44)	<0.0001
**Left Atrium**				
LA 3Ch dimension (mm)	40 (36–49)	39 (37–44)	39 (367–46)	0.3726
LAA max 4Ch (cm^2^)	32 (24–35)	28 (24–33)	28 (24–34)	0.2119
LAL max 4Ch (mm)	71 (57–79)	62 (57–69)	64 (57–72)	0.0242
LAA min 4Ch (cm^2^)	22 (18–27)	17 (14–22)	18 (15–25)	0.0090
LAL min 4Ch (mm)	61 (51–74)	53 (44–59)	54 (47–63)	0.0030
LAA max 2Ch (cm^2^)	26 (24–34)	25 (23–28)	25 (23–31)	0.1244
LAL max 2Ch (mm)	63 (58–72)	61 (56–65)	61 (57–68)	0.1397
LAA min 2Ch (cm^2^)	19 (16–27)	16 (13–20)	17 (13–22)	0.0075
LAL min 2Ch (mm)	53 (48–64)	50 (43–54)	51 (45–58)	0.0263
LAV max (mL)	118 (79–135)	94 (78–120)	97 (78–125)	0.2665
LAV min (mL)	67 (44–92)	44 (34–62)	47 (36–74)	0.0089
LAEF (%)	38 (26–43)	53 (42–60)	48 (37–57)	0.0001
LAVI (mL/m^2^)	58 (36–69)	49 (41–62)	51 (41–65)	0.4027
**LVOT and mitral apparatus**				
LVOT (mm)	21 (17–24)	15 (10–17)	16 (13–20)	<0.0001
Ao (mm)	26 (23–28)	23 (21–25)	23 (21–26)	0.0025
LVOT/Ao	0.86 (0.77–0.89)	0.65 (0.45–0.75)	0.72 (0.58–0.83)	<0.0001
AML (mm)	26 (23–30)	24 (22–26)	25 (22–27)	0.1925
PML (mm)	14 (13–16)	14 (13–16)	14 (13–16)	0.8585
AML/LVOT	1.27 (1.08–1.45)	1.56 (1.33–2.5)	1.44 (1.26–2.07)	<0.0001
LVOTO visible in 3Ch	0%	20 (32%)	20 (23%)	0.0010

Parameters are expressed as median (interquartile range). List of abbreviations: CP—classic phenotype, NCP—nonclassic phenotype, BSA—body surface area, BMI—body mass index, HCM risk score—hypertrophic cardiomyopathy European Society of Cardiology risk score (2014), LVEDV—left ventricular end diastolic volume, LVESV—left ventricular end systolic volume, SV—stroke volume, CO—cardiac output, LV mass—left ventricular mass, LVMT—left ventricular maximal thickness, RVMT—right ventricular maximal thickness, PER—peak ejection rate, PET—peak ejection time, PFR—peak filling rate, PFT—peak filling time, LGE%—late gadolinium enhancement expressed as the percent of myocardial mass, LGE volume—late gadolinium enhancement volume, LVEDVI—left ventricular end diastolic volume index, LVMI—left ventricular mass index, wall to volume ratio—left ventricular maximal thickness to left ventricular end diastolic volume ratio, IMWT—indexed left ventricular maximal wall thickness, MCF—myocardial contraction fraction, LVGFI—left ventricular global function index, LA 3Ch dimension—transverse left atrial dimension in three-chamber view, LAA max—maximal left atrial area, LAL max—maximal left atrial length, 2Ch—two-chamber view, 4Ch—four-chamber view, LAA min—minimal left atrial area, LAL min—minimal left atrial length, LAV max—maximal left atrial volume, LAV min—minimal left atrial volume, LAEF—left atrial emptying fraction, LAVI—maximal left atrial volume index, LVOT—left ventricular outflow tract diameter, Ao—aortic valve diameter, AML—anterior mitral leaflet length, PML—posterior, and LVOTO visible in 3Ch—presence of flow void and obstruction visible in left ventricular outflow tract in cine three-chamber image.

**Table 2:** Left ventricular segmental mean myocardial thickness and segmental distribution of late enhancement areas—comparison between the nonclassic and classic phenotype.

LV Segment	Mean Thickness			LGE Presence		
	NCP	CP	*p* Value	NCP	CP	*p* Value
**1**	14	12	0.0160	10 (39%)	18 (29%)	0.3862
**2**	14	14	0.5491	18 (69%)	30 (48%)	0.0732
**3**	14	14	0.7420	19 (73%)	21 (34%)	0.0008
**4**	13	12	0.2454	8 (31%)	11 (18%)	0.1754
**5**	12	12	0.4928	10 (39%)	13 (21%)	0.0884
**6**	11	11	0.3750	6 (23%)	5 (8%)	0.0520
**7**	12	10	0.0176	10 (39%)	11 (18%)	0.0375
**8**	13	10	0.0027	15 (58%)	21 (34%)	0.0381
**9**	15	11	0.0004	15 (58%)	27 (44%)	0.2255
**10**	14	11	0.0031	13 (50%)	15 (24%)	0.0177
**11**	13	10	0.0346	7 (27%)	9 (15%)	0.1686
**12**	11	10	0.1372	3 (12%)	5 (8%)	0.6050
**13**	13	10	0.1222	5 (19%)	8 (13%)	0.4453
**14**	11	10	0.0621	2 (8%)	9 (15%)	0.3772
**15**	11	10	0.0396	4 (15%)	7 (11%)	0.5962
**16**	12	10	0.1244	3 (12%)	5 (8%)	0.6050

## Data Availability

The data presented in this study are available upon request from the corresponding author.

## Supplementary Materials

The following supporting information can be downloaded at: https://www.mdpi.com/article/10.3390/diagnostics12051104/s1, Table S1: Clinical characteristics of the study groups, Table S2: Availlable follow-up results.
