# Supplementary material for: Comparison of Nonclassic and Classic Phenotype of Hypertrophic Cardiomyopathy Focused on Prognostic Cardiac Magnetic Resonance Parameters: A Single-Center Observational Study
Source: Diagnostics (Basel). 2022 Apr 28;12(5):1104. doi: 10.3390/diagnostics12051104 (PMC9139797; doi:10.3390/diagnostics12051104)
Supplement: Supplementary file 1 [file diagnostics-12-01104-s001.zip › diagnostics-1684625-supplementary.pdf]

diagnostics

MDPI

Supplementary Material

Table S1. Clinical characteristics of the study groups

Parameter	NCP	CP	N (NCP)	N (CP)	P value
Atrial fibrillation	8 (30,8%)	9 (14,5%)	26	62	0,142
Asthma	0 (0%)	1 (2,1%)	21	47	0,677
Chest pain CSS: 1	6 (28,6%)	14 (29,8%)	21	47	0,787
Chest pain CSS: 2	0 (0%)	1 (2,1%)	21	47	0,787
No coronary lesions	1 (4,8%)	7 (14,9%)	21	47	0,429
Diabetes	7 (33,3%)	5 (10,4%)	21	48	0,049
Invasive coronarography	4 (19,1%)	10 (21,3%)	21	47	0,909
Dyspnea	6 (28,6%)	10 (21,3%)	21	47	0,729
Palpitations	5 (23,8%)	15 (32,6%)	21	46	0,658
Peripheral atherosclerosis	1 (4,8%)	2 (4,2%)	21	48	0,596
Hypertension	14 (66,7%)	25 (53,2%)	21	47	0,440
Renal insufficiency	2 (9,5%)	1 (2,1%)	21	47	0,464
Smoking	5 (23,8%)	8 (17%)	21	47	0,746
Heart failure symptoms	10 (47,6%)	7 (14,6%)	21	48	0,009
Syncope	5 (23,8%)	11 (23,4%)	21	47	0,785
Post revascularization	3 (14,3%)	1 (2,1%)	21	47	0,158
COPD	3 (14,3%)	0 (0%)	21	47	0,044
Stroke	1 (4,8%)	0 (0%)	21	47	0,677
Myocardial infarction	2 (9,5%)	4 (8,5%)	21	47	0,744
Family history of HCM	1 (5%)	11 (24,4%)	20	45	0,129
Diastolic dysfunction in echo	11 (61,1%)	17 (37%)	18	46	0,141

Parameters are expressed as numbers (percentage). List of abbreviations: CCS – Canadian Cardiac Society scale, COPD – chronic obstructive pulmonary disease, CP - classic phenotype of HCM, HCM – hypertrophic cardiomyopathy, N – number of patients with data available, NCP – nonclassic phenotype of HCM

Table S2. Availlable follow-up results

	NCP	CP	P value
	N = 26	N = 62	
Pulmonary vein ablation	1 (3,9%)	2 (3,2%)	0,61886
ICD implantation	8 (30,8%)	10 (16,1%)	0,20631
ICD discharges	3 (37,5%)	0 (0%)	0,13756
Hospitalizations	5 (19,2%)	11 (17,7%)	0,8905
Death	2 (7,7%)	1 (1,6%)	0,42947

Parameters are expressed as numbers (percentage). List of abbreviations: ICD – implantable cardioverter-defibrillator
